# Electroencephalography measures of relative power and coherence as reaching skill emerges in infants born preterm

**DOI:** 10.1038/s41598-021-82329-7

**Published:** 2021-02-11

**Authors:** Ryota Nishiyori, Ran Xiao, Douglas Vanderbilt, Beth A. Smith

**Affiliations:** 1 Division of Research on Children, Youth, and Families, Children’s Hospital Los Angeles, Los Angeles, USA; 2 School of Nursing, Duke University, Durham, USA; 3 Department of Pediatrics, Division of General Pediatrics, Keck School of Medicine, University of Southern California, Los Angeles, USA; 4 Department of Pediatrics, Keck School of Medicine, University of Southern California, Los Angeles, USA; 5 Developmental Neuroscience and Neurogenetics Program, The Saban Research Institute, Los Angeles, USA

**Keywords:** Motor cortex, Developmental disorders

## Abstract

Electroencephalography (EEG) measures of relative power and coherence are associated with motor experience in infants with typical development, but these relationships have not been assessed in infants born preterm. The goal of our study was to investigate the changing patterns of relative power and coherence in the alpha band during resting state EEG in infants born preterm as they developed the skill of reaching. We collected monthly longitudinal data from fourteen infants born preterm between the adjusted ages of 56 and 295 days for a total of 37 sessions of EEG data. Alpha band power at motor cortices and cross-regional connectivity do not present consistent changing trends at the group level in infants born preterm. Individual level analysis reveals that infants born preterm are a heterogeneous group with subtypes of neural function development, some presenting similar changing trends as observed in the typically developing group while others present atypical patterns. This may be linked to the variability in developmental outcomes in infants born preterm. This study was a critical first step to support EEG as a potential tool for identifying and quantifying the developmental trajectories of neuromotor control in infants born preterm.

## Introduction

Compared to infants born at term, infants born preterm are at risk for impairments in fine and gross motor skills^1^ and speaking and writing^2^. Basic neuroscience principles and laboratory data support that early intervention can positively impact experience-dependent plasticity, thus improving the trajectory of motor development^3^. A recent review supports the importance of implementing early interventions, however only a few interventions have shown positive effects on motor outcomes and only in the short term (see^4^ for review). A contributing factor to the low number of successful interventions and a prominent challenge in the field is our lack of ability to quantitatively assess impaired neuromotor control during infancy. Establishing a quantitative assessment of infant neuromotor control would provide a measure to track infants born preterm who are at risk for neuromotor impairments, thus allowing clinicians to provide early and targeted interventions and assess outcomes.

To date, there are no scales of infant development that can, with high sensitivity and specificity, identify and quantify impaired neuromotor development within the first months of life. For example, the Alberta Infant Motor Scale (AIMS), using a cutoff of below the 10th percentile at 4 months of age for infants born at less than 36 weeks of gestation, yielded a 77.3% sensitivity and 81.7% specificity to predict abnormal from normal classification at 18 months^5,6^. Prechtl’s General Movements Assessment (GMA), while highly sensitive and specific (98% and 91%) at predicting cerebral palsy^7^ (CP), is less accurate in predicting poor neuromotor outcomes more broadly. When infants born at less than 30 weeks’ gestation showed abnormal movements at 1 month of corrected age, GMAs produced a sensitivity of 100% and specificity of 42% to predict moderate to severe motor impairment at 2 years of age. When the same infants were tested at 3 months corrected age, sensitivity was 70% and specificity at 85% to predict moderate to severe motor impairments at 2 years of age^8^. Both the AIMS and GMA, two widely used assessments, are not highly accurate at predicting later motor impairments, nor do they quantify impaired neuromotor development early in life.

Electroencephalography (EEG) shows potential to be used to quantify neuromotor development. Previous studies have shown that differences in mu rhythm are associated with the acquisition of functional motor skills and the role of motor experience. Mu rhythms are similar in frequency range with the alpha range but specifically arise from the motor cortex and were more strongly suppressed in the 7 to 9 Hz range when infants observed a familiar motor skill compared to an unfamiliar one^9^. Moreover, Xiao and colleagues^10^ found that in the weeks leading up to the onset of crawling, infants exhibited increased resting state power in the alpha band and spatio-spectral patterns centered upon the central areas, which were not observed in lower bands such as delta and theta^10^. Xiao and colleagues^11^ examined infants as young as 1 month and showed that resting state alpha band power increases with age, with a marked “bump” at 7 months of age. The researchers expanded upon the relationship between mu rhythm development and the emergence of motor skills, measured by reaching skill level and motor scores on the Bayley Scales of Infant and Toddler Development, 3rd edition^12^ (BSID-III).

EEG also provides a way of quantifying the existing functional connections between brain regions (coherence), which has been shown to change as infants learn functional motor skills. Bell and Fox^13^ examined the changes in cortical development as a function of crawling experience in 8-month-old infants and showed that the onset of locomotion is associated with changes in resting state cortical organization. Infants with 1 to 8 weeks of crawling experience showed greater coherence between frontal-parietal, parietal-occipital, and frontal-occipital regions compared to same-aged non-crawlers and experienced crawlers (> 9 weeks). Similarly, Corbetta et al. investigated a similar question, but related to walking experience^14^. The researchers found a similar association between motor experience and changes in resting state coherence. That is, novice walkers exhibited the highest levels of coherence compared to same-aged non-walkers and experienced walkers. All infants were 12 months of age; the emergence of walking occurred at different ages and its emergence was associated with patterns of cortical reorganizations and changes in brain connectivity^14^. Indeed, these studies have shown that in infants with typical development (TD), motor experience and the emergence of functional motor skills are reflected in changes in resting state EEG measures of relative power and coherence.

To date, most research has focused on examining alpha band activities to understand its relationship with typical neuromotor development. Our previous research has shown that infants with TD exhibit changes in resting state relative power and coherence within the alpha band as reaching skill emerged^11^. Our next step was to examine if infants born preterm showed similar neurodevelopmental patterns. Thus, our objective here was to investigate the relationship between changes in alpha band activities, via relative power and coherence, and motor skills in infants born preterm. To characterize the longitudinal development of neural function, we took monthly measurements of EEG as arm reaching skill developed across the early months of life. We studied the developmental changes in connections between brain regions and modeled the relationship between changes in coherence in the alpha band and age, and motor development scores (reaching skill level, fine and gross motor scores from the BSID-III^12^ in infants born preterm. Finally, we used linear regression fitting for individual participants to compare variations across individual developmental trends between infants born preterm (this sample) and infants with typical development (previous sample).

## Results

We collected monthly EEG measurements and synchronous video recordings from fourteen infants born preterm (adjusted ages between 56 and 295 days), from which we computed various EEG metrics to comprehensively evaluate characteristics of neuromotor development in infants born preterm. We first calculated power spectral density to study the spectral and spatial profiles of infant alpha band from the motor cortices. Next, brain connectivity was captured by alpha band coherence of EEG channel pairs placed at different brain regions. For all EEG metrics, we studied their longitudinal changes along age (i.e., adjusted age) and various motor development scores (reaching skill level determined by analyzing synchronous videos, raw fine and gross motor scores from the Bayley Scales of Infant Development-III). Table 1 provides participant characteristics from at birth.

**Table 1:** Participant characteristics at birth.

Participant	Gender	Gestational age	Weight (kg)	Length (cm)
1	M	23 weeks 2 days	1.79	–
2	M	28 weeks 5 days	1.22	36.83
3	M	24 weeks 4 days	1.59	38.10
4	M	31 weeks 6 days	1.81	–
5	M	31 weeks 1 days	1.79	40.64
6	M	31 weeks 4 days	1.60	38.10
7	F	26 weeks 1 days	0.74	–
8	M	25 weeks 0 days	0.62	–
9	F	30 weeks 5 days	1.33	38.99
10	F	30 weeks 4 days	1.45	38.10
11	F	28 weeks 4 days	1.45	–
12	M	32 weeks 0 days	1.77	41.91
13	F	30 weeks 3 days	1.53	39.37
14	M	24 weeks 2 days	0.65	30.99

A (−) indicates no data for participant.

### EEG spectro-spatial patterns along monthly age

**Figure 1:**
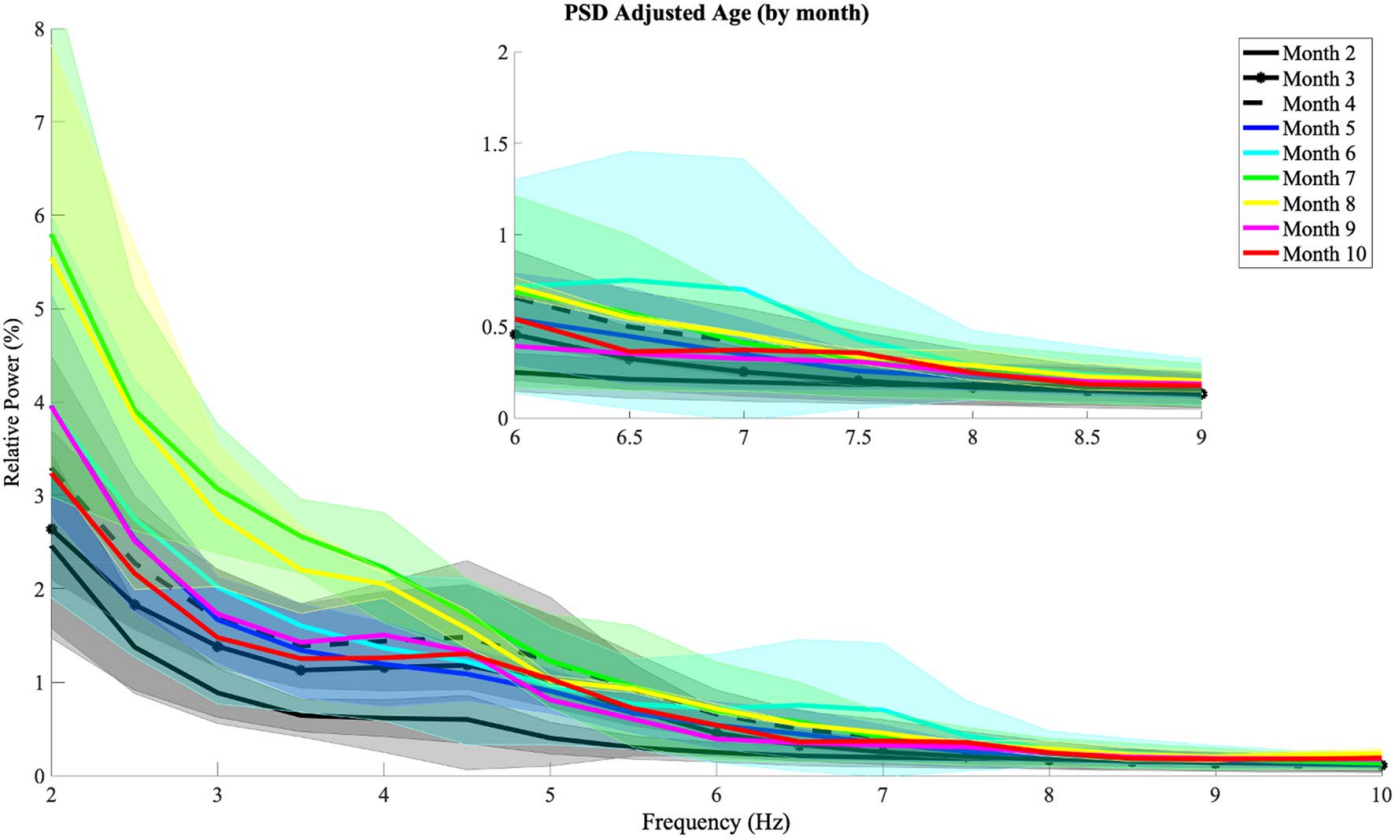
Monthly spectral profiles (power spectral densities, PSD) from motor cortices, channels C3, Cz, and C4. Shaded areas represent standard deviation for each month group (adjusted age). Inset provides a zoom-in view of spectral profiles in the 6–9 Hz.

The spectro-spatial patterns in EEG were first examined against monthly age to quantitatively evaluate how alpha band power changes along age and corresponding progression in cortical patterns. Monthly age was calculated based on the infant’s adjusted age in days at the visit. We grouped and averaged the spectral profiles with the same monthly age (Month 2 = ages 56 to 83, Month 3 = ages 84 to 111, Month 4 = ages 112 to 139, Month 5 = ages 140 to 167, Month 6 = 168 to 195, Month 7 = 196 to 223, Month 8 = 224 to 251, Month 9 = 252 to 279, Month 10 = 280 to 307 adjusted days). Spectral profiles (in relative power) of the 0 to 30 Hz range from the motor cortices were grouped by their respective monthly age. Figure 1 presents monthly spectral profiles in the 2 to 10 Hz range to best emphasize changes in the frequency range of interest).

Each line represents the average spectral profile across all sessions from one monthly age color coded from cool to warm with increasing age. The shaded area shows the region with one standard deviation of sessions of the same monthly age. Based on visual inspection, notable changes can be observed within the 3 to 6 Hz band, but not in the 6 to 9 Hz (alpha) band. Although lowest spectral profiles can be observed in our youngest groups (black lines), neither alpha nor lower frequency bands show consistent changing patterns in relative power.

**Figure 2:**
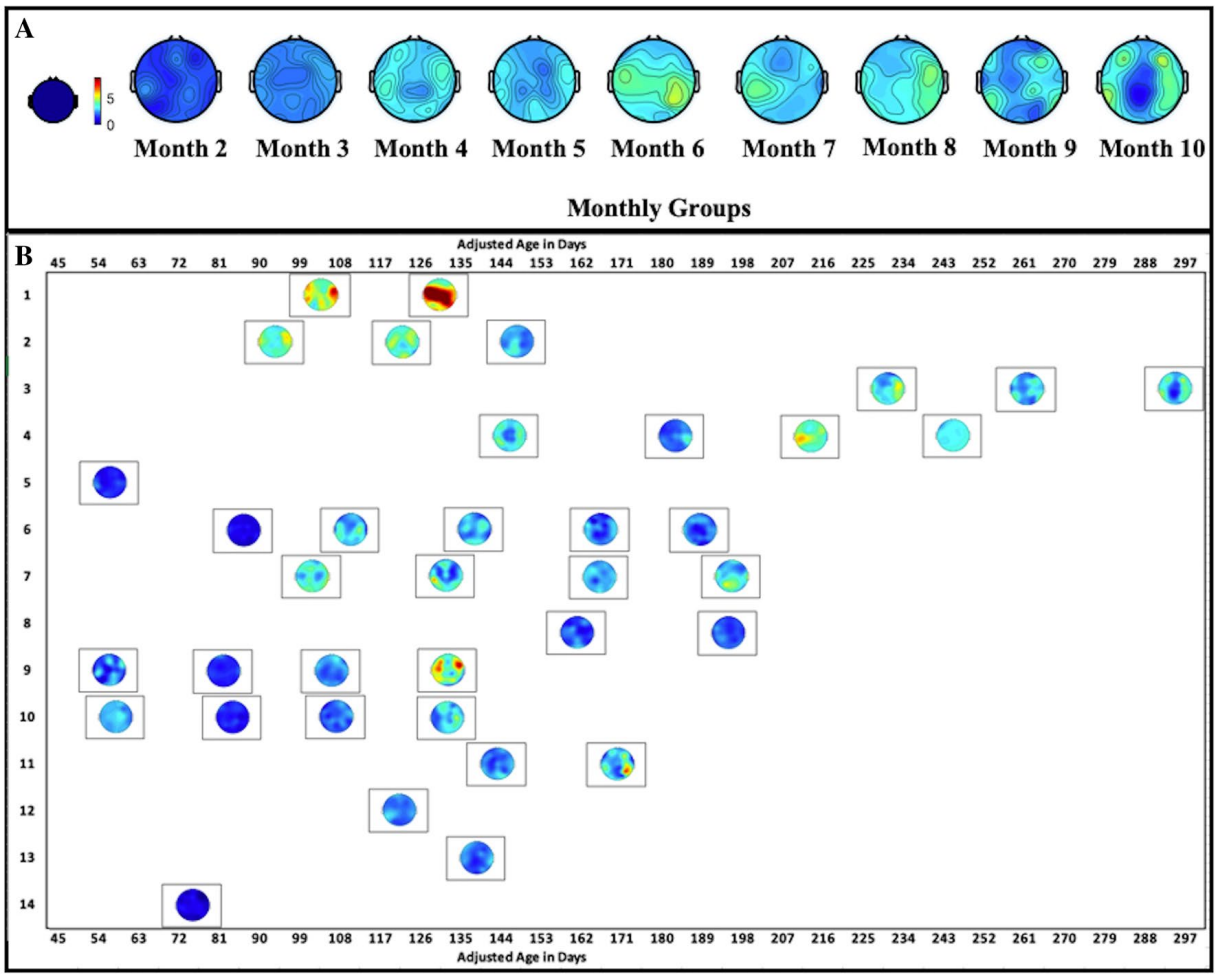
(**A**) Individual topography maps of alpha band power (6–9 Hz) for all sessions. Topographies of each infant's (y-axis) visits are plotted along adjusted age in days (x-axis). (**B**) Topographies of alpha band power for each month group (adjusted age). Warmer colors represent a higher relative power.

Changes in spatial patterns of alpha band power were visually evaluated through topographic maps grouped by monthly age in Fig. 2A.

Each plot represents the average alpha topographic map based on one monthly age. The maps present a general increasing pattern of cortical activity in alpha band along age, especially between early months (2 and 3) and later months (4 and after). With increasing age, alpha band power emerges, and the emergence and enlargement of alpha power can mostly be observed within or near the regions of the motor cortices.

### Alpha band power and spatial patterns along age in adjusted days

Besides developmental changes in alpha band power grouped at the monthly level, the following results present changes in spectro-spatial patterns of alpha band power at the individual-session level with a daily age resolution. Figure 2B shows the topographic maps of each individual session plotted against age in adjusted days. Based on visual inspection, individual level variability in the changing of alpha power can be observed. Most participants with multiple EEG recordings present general increasing trends (although some are not monotonic) comparing earlier to later sessions. The changes from cooler to warmer colors surrounding the central cortical areas can be seen in a subset of infants, representing an increase of alpha band power with age, while others show mixed patterns.

**Figure 3:**
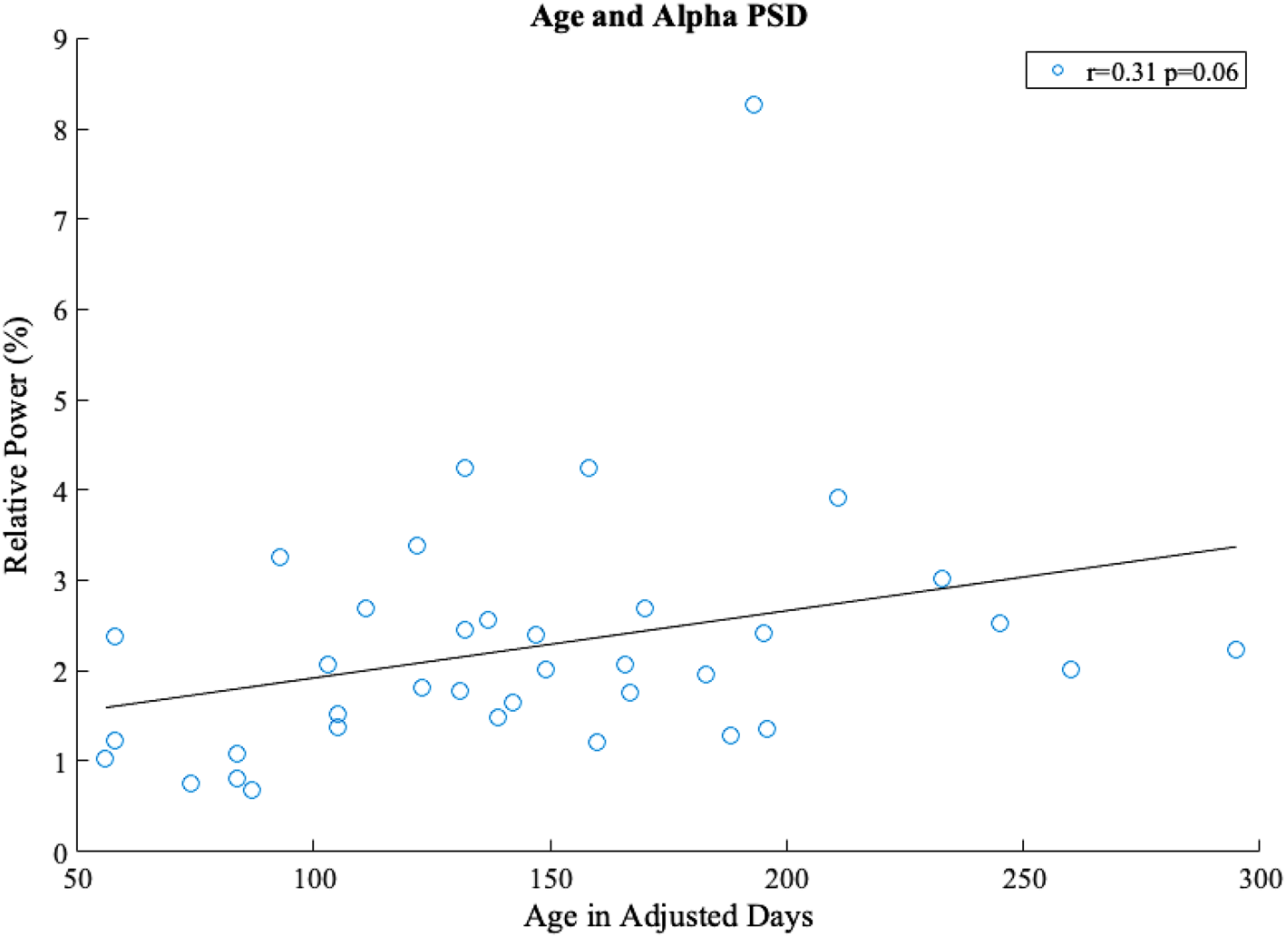
Correlation of alpha band power (6–9 Hz) from motor cortices (C3, Cz, and C4) with adjusted age in days. Each circle represents the alpha band power from an individual session. The bold line shows the least-squares fitted line along age. The *r* and *p* values are the results from the correlation test.

A scatter plot of alpha band power from individual sessions against corresponding age in adjusted days is shown in Fig. 3.

It reveals a potentially increasing trend between alpha band power and age. The Pearson correlation test showed that these variables were not significantly correlated (r = 0.31, *p* = 0.06). Furthermore, we conducted a likelihood ratio test with two LMM models, with and without subject variation as the random effect to assess the impact of repeated measures from subjects. The test demonstrated that there was no significant impact of subject variation on the changes of alpha activities along age (χ^2^ = 3.31, *p* = 0.07).

To further validate the correlation between alpha band power and age, a bootstrapping approach was adopted to generate a null distribution of correlations between pseudo alpha power with randomly shuffled frequencies and age, which was compared with the true correlation. Using the two-sided Wilcoxon rank sum test, the correlation between alpha band power and age is not significantly different from random (*p* = 0.24).

### Comparison of variance in developmental heterogeneity

This subsection provides insights into developmental heterogeneity in in our current sample of infants born preterm and its comparison to our previously collected data from infants with TD^11^. Data from participants with at least two EEG recording sessions in both groups were selected for the analysis, resulting in 10 participants in the preterm group and 18 participants in the TD group. Linear regression fitting was performed for each participant with various fitting metrics extracted to capture variations across individual developmental trends. Variances in gradients and intercepts were first calculated to evaluate cross-participant differences within each group. In addition, developmental changes within each participant were accounted for by the variance in fitting errors. The variances of three fitting metrics are presented in Table 2. The preterm group shows higher variances in all three metrics compared with infants with TD.

**Table 2:** Gradience, intercept, and fitting error values from best-fitted line between alpha-motor relative powers and age at respective visits.

PT				TD			
Participant	Gradient	Intercept	Fitting error	Participant	Gradient	Intercept	Fitting error
PT1	0.12	− 13.94	3.97E−15	TD2	2.10E−03	1.78	0.79
				TD3	1.27E−02	0.33	1.47
PT2	3.08E−03	1.46	1.38	TD4	− 5.38E−03	2.56	0.27
				TD5	− 2.50E−02	8.42	4.44E−15
PT3	3.67E−02	−3.56	8.34E−07	TD9	− 2.36E−02	5.64	0.58
				TD11	− 4.75E−03	3.10	0.00
PT4	− 2.21E−02	5.57	0.64	TD12	1.10E−02	1.89	0.75
				TD13	7.74E−02	− 7.69	1.99E−15
PT6	− 1.16E−02	5.46	0.55	TD14	− 2.43E−02	7.20	0.99
				TD15	1.99E−02	0.12	4.97E−16
PT7	6.60E−03	1.40	1.39	TD17	− 8.84E−03	4.72	4.44E−16
				TD18	4.78E−02	− 0.98	0.67
PT8	8.29E−04	1.68	1.70	TD20	1.35E−02	1.24	0.76
				TD21	5.14E−02	− 2.18	1.33E−15
PT9	4.49E−03	1.41	0.33	TD22	− 4.37E−03	4.58	1.93
				TD23	− 1.86E−02	5.64	1.50
PT10	3.94E−03	0.58	1.69E−07	TD24	3.67E−02	− 1.10	8.88E−16
				TD25	− 3.97E−02	7.29	1.60E−15
PT11	3.95E−02	− 1.72	1.45				
Variance	1.53E−03	31.09	0.46	Variance	9.53E−04	16.04	0.38
Min	− 0.02	− 13.94	3.97E−15	Min	− 0.04	− 7.69	0.00
Max	0.12	5.57	1.70	Max	0.08	8.42	1.93
Average	0.02	− 0.17	0.74	Average	0.01	2.36	0.54
Stdev	0.04	5.58	0.68	Stdev	0.03	4.00	0.62

Variance, minimum, maximum, average, and standard deviation were calculated within the subset of infants who had two or more visits shown here.

### Brain connectivity along age and motor experience

**Figure 4:**
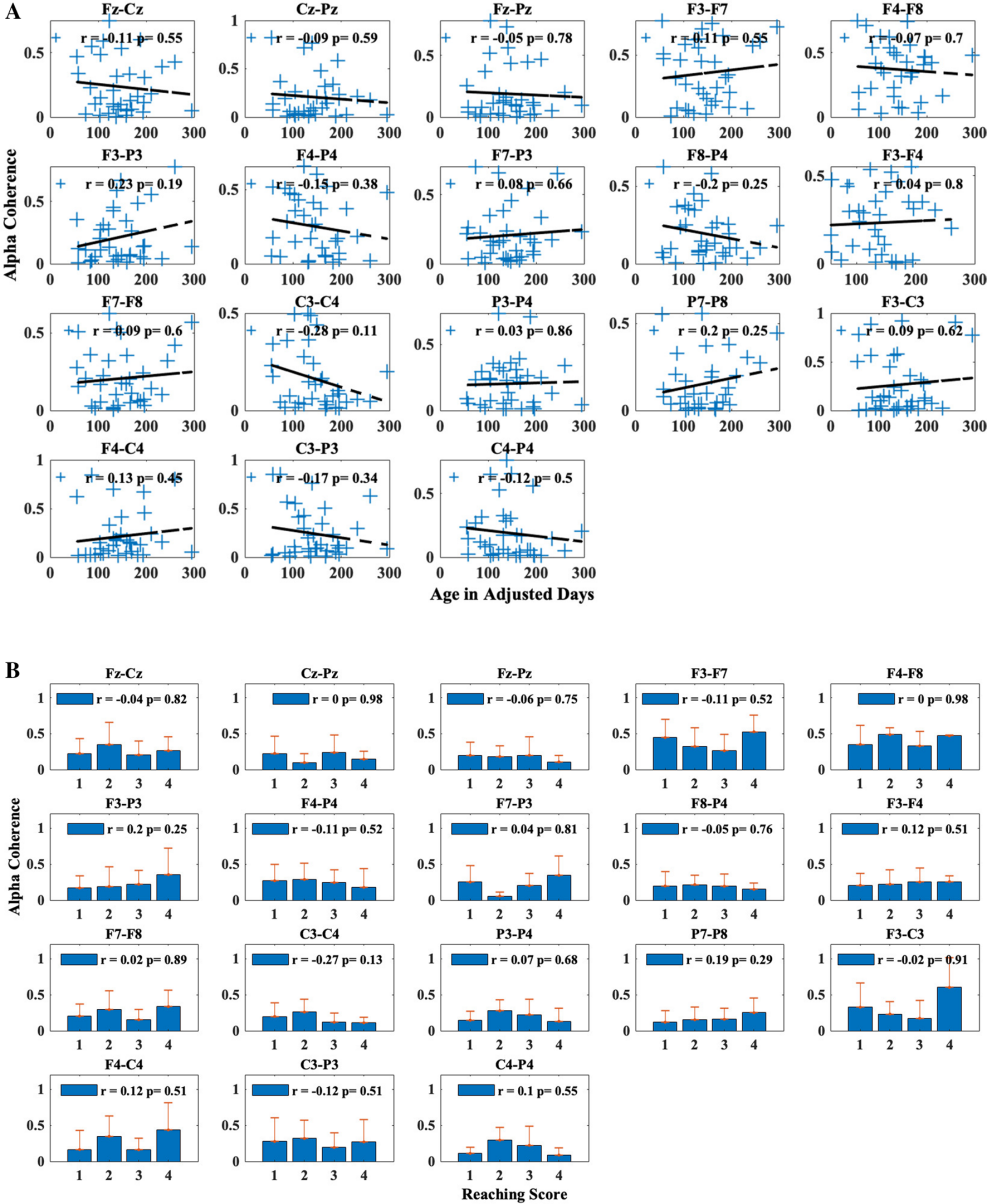
Change in mean alpha (6–9 Hz) coherence (**A**) along adjusted age in days and (**B**) across reaching skill levels for each electrode pair. Within each scatter subplot, blue crosses represent mean alpha coherence for each session and dashed lines represent the least-squares fitted lines. With each bar subplot, bars represent mean alpha coherence for sessions with the same reaching skill level with standard deviation error bars on top. The *r* and *p* values are the results from the correlation test.

Brain connectivity was evaluated by calculating the mean-squared coherence in the alpha band for 18 electrode pairs covering frontal, motor, and parietal brain regions, chosen a priori based on previous research^11^. In Fig. 4A, the changes in alpha coherence are plotted against age in adjusted days.

**Figure 5:**
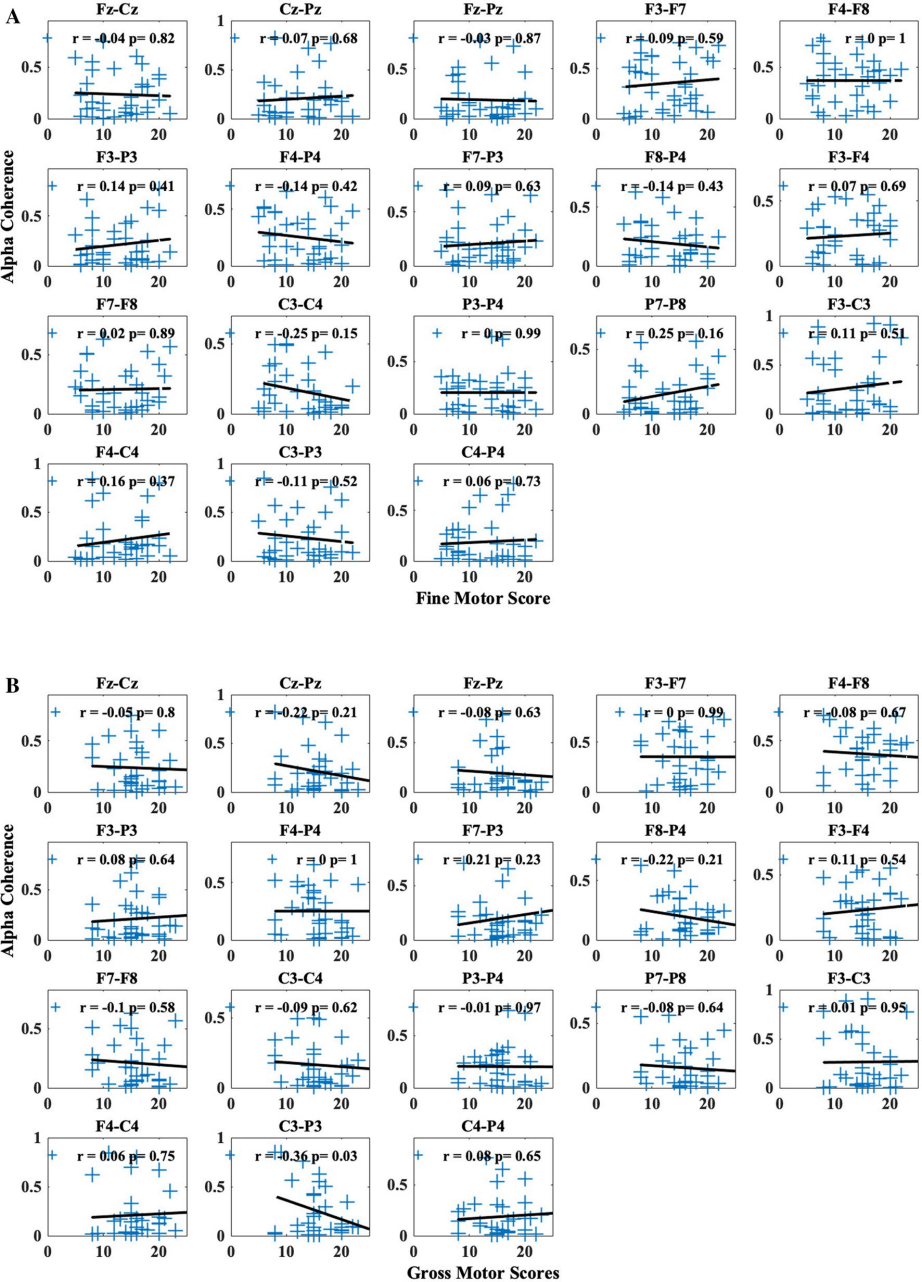
Change in mean alpha coherence along (6–9 Hz) (**A**) fine motor and (**B**) gross motor scores from Bayley Scales of Infant and Toddler Development, 3rd edition for each electrode pair. Within each subplot, blue crosses represent mean alpha coherence for each session and dashed lines represent the least-squares fitted lines. The *r* and *p* values are the results from the correlation test.

There is no clear significant pattern of change in coherence as age increases (*p* > 0.05). Similarly, reaching skill levels did not show a pattern that would significantly correlate (*p* > 0.05) with changes in alpha coherence across the electrode pairs (Fig. 4B). Finally, both fine motor (Fig. 5A) and gross motor (Fig. 5B) scores are scattered and show no significant trend with any of the electrode pairs.

Table 3 provides the results of the likelihood ratio tests (chi-square and *p* values) of our predictors and relationship with changes in alpha coherence.

**Table 3:** Likelihood ratio tests for changes in mean alpha coherence across adjusted age (days), reaching skill level, fine motor scores, and gross motor scores, for 18 electrode pairs. Alpha value of 0.05/18 was selected for the statistical tests.

Electrode pair	Adjusted age		Reaching scores		Fine motor scores		Gross motor scores	
	χ^2^	p	χ^2^	p	χ^2^	p	χ^2^	p
Fz–Cz	0.37	0.54	0.08	0.78	0.00	0.97	0.04	0.84
Cz–Pz	0.15	0.70	0.01	0.93	0.22	0.64	2.09	0.15
Fz–Pz	0.36	0.55	0.00	1.00	0.16	0.69	0.51	0.48
F3–F7	0.39	0.53	0.44	0.51	0.26	0.61	0.00	0.98
F4–F8	0.29	0.59	0.00	0.96	0.09	0.76	0.09	0.76
F3–P3	1.19	0.28	1.72	0.19	0.60	0.44	0.14	0.71
F4–P4	0.90	0.34	0.68	0.41	0.58	0.45	0.02	0.89
F7–P3	0.23	0.63	0.00	0.97	0.01	0.92	0.02	0.89
F8–P4	1.41	0.24	0.11	0.74	1.36	0.24	2.46	0.12
F3–F4	0.07	0.80	0.47	0.49	0.18	0.67	0.41	0.52
F7–F8	0.29	0.59	0.01	0.90	0.02	0.90	0.38	0.54
C3–C4	2.83	0.09	1.89	0.17	2.37	0.12	0.61	0.43
P3–P4	0.03	0.86	0.19	0.66	0.00	0.99	0.00	0.97
P7–P8	0.65	0.42	0.33	0.56	0.71	0.40	0.41	0.52
F3–C3	0.26	0.61	0.02	0.90	0.01	0.90	0.00	0.99
F4–C4	0.60	0.44	0.58	0.45	0.83	0.36	0.03	0.85
C3–P3	0.98	0.32	0.42	0.52	0.44	0.50	4.50	0.03
C4–P4	0.48	0.49	0.88	0.35	1.07	0.30	0.10	0.75

We adjusted the alpha level to 0.05/18 to address the issue of multiple comparisons. Based on this adjusted alpha level, the likelihood ratio tests did not detect any significant relationships between our predictors and coherence values for any of the 18 electrode pairs. Figures 4 and 5 depict the relationship between the predictor (age, reaching skill level, BSID-III fine and gross motor raw subscale scores) with each of the electrode pairs with a Pearson correlation value (r) with a least-squares fitted line to represent the changing trend.

## Discussion

The goal of our study was to examine resting state relative power and coherence in the alpha band in infants born preterm across the time period when reaching was emerging. Neurophysiological metrics (alpha band power and coherence) from longitudinal resting state EEG recordings in infants born preterm holds important information on changes in neural function in this high-risk group and profiles their change (or lack of change, in some cases) in patterns along maturation and motor skill development. The preterm group did not show a consistent pattern of change as we previously saw in a group of infants with TD. This study was a critical first step to support EEG as potential tool for identifying and quantifying the developmental trajectories of neuromotor control in infants born preterm.

In this study, we evaluated alpha band power longitudinally as reaching was emerging in infants born preterm. Overall, we did not observe an increasing pattern in alpha band power when spectral profiles were grouped by monthly age (Fig. 1). Relative power from individual sessions potentially showed a pattern of increase along adjusted age in days that did not reach statistical significance (Fig. 3). Topographies of alpha band power along monthly age show a pattern for the emergence of alpha band power, centering around the motor areas (Fig. 2B). Individual topographies along adjusted age in days reveal diverse changing patterns in alpha band power, with a subset of the infants born preterm in our sample showing increases in alpha band power and others without a cohesive changing pattern (Fig. 2A).

We also examined the changes in coherence and their associations with predictors (age, reaching skill level, fine motor scores, and gross motor scores). Using Pearson correlation, we examined the linear relationship between changes in coherence and age and motor development scores. Although coherence values from electrode pairs, such as C3–C4 and C3–P3, showed certain levels of changing trends based on age (*r* = − 0.28 and − 0.17, respectively) or various motor development scores (reaching skill level: *r* = − 0.27 and − 0.12; fine motor: *r* = 0.25 & − 0.11; gross motor: *r* = − 0.09 and − 0.36, for each electrode respectively), these relationships did not reach statistical significance using likelihood ratio tests with LMM for each predictor after adjustment for multiple comparisons (adjusted age: *p* = 0.09 and 0.32; reaching skill level: *p* = 0.17 and 0.52; fine motor: *p* = 0.12 and 0.50; gross motor: *p* = 0.43 and 0.03, respectively) (Table 3).

Overall, our results differ from those seen in a previous study with infants with TD. Our results in the previous study revealed that infants with TD show an increase in resting state alpha band power during the first 7 months of age^11^. In the present study, alpha activities, when grouped by monthly age, showed no marked increase in relative power. At an individual level, however, a subset of infants showed increase in alpha band relative power, while others did not. This indicates that some infants born preterm might follow a similar developmental path to infants with TD, while others do not, and this can potentially be discerned using EEG before motor skill differences are apparent. Furthermore, our previous study showed that connectivity between frontal-parietal regions increased as gross motor scores increased and interhemispheric connectivity in motor cortices decreased as fine motor scores increased in infants with TD^11^. In the present study, infants born preterm did not exhibit significant relationships at a group level between changes in alpha coherence and age, reaching skill level, or fine and gross motor scores. Thus, group-level analyses may not capture the individual differences in infants born preterm. This is consistent with the variability in developmental outcomes for infants born preterm, some end up with diagnoses of developmental disabilities while others do not.

Our study focused on the emergence of alpha band power during a time when infants are becoming skilled at reaching. As such, our objective centered upon the alpha band or mu-rhythm, which has been associated with the development of functional motor skills in infants with TD^15^. The infants born preterm in our sample did not show a clear increasing trend in both reaching skill level and fine and gross motor scores in the BSID-III, and moreover they did not show changes in the alpha band at a group level. Although the changes in behavior based on these same scores were reflected at the group level in EEG measures in infants with TD^11^, this was not true here in infants born preterm. Infants in our sample, as shown in Table 1, represent the variability typically seen in this population.

This suggests two possibilities: (1) infants born preterm are a heterogenous group, some of whom look similar to infants with TD while others do not, and/or (2) changes may be observed in other frequency bands. We took a bootstrapping approach and demonstrated that infants born preterm did not exhibit a significant alpha-band specific correlation with age. This further suggests that infants born preterm may show emergence of relative power at different frequency bands and points in time, unlike the commonly observed changes in alpha-band power in infants with TD.

In regard to other frequency bands, previous studies have shown that infants born preterm exhibit increase in power at lower frequency bands, such as delta (0.5 to 4 Hz) or theta (4 to 8 Hz) bands compared to control or infants born near term^16,17^. Suppiej and colleagues have shown that infants born extremely low gestational age (ELGA) have higher relative power in the delta (0.5 to 4 Hz) band compared to healthy late preterm infants born between 34 to 35 weeks of gestational age (control). The study also found that other frequency bands such as theta and alpha were lower in the ELGA group compared to the control group. Such shift in relative power bands has been shown in other studies investigating preterm infants and newborn infants^16,17^. Similarly, in our dataset, we did not see an increase in alpha band power from a sample that includes infants that would fall into the category of extremely low gestational age, as shown in the aforementioned study^17^. Thus, future research should examine more closely the emergence of relative power in frequency bands such as theta, and its relationship to age and motor development scores.

Our study results were able to shed light on the heterogenous group within infants born preterm—patterns of change similar and dissimilar to infants with TD. While the group of infants born preterm did not show a significant increase in relative power, a subset of infants born preterm showed an increase in relative power in the alpha band similar to infants with TD. Further research is warranted in infants born preterm to characterize the relationships between brain function and motor development in infants born preterm who showed change in EEG patterns similar and dissimilar to infants with TD. By doing so, we will be able to further develop the use of EEG as a useful tool to quantify neuromotor development early in life, before differences in motor skill development can be accurately assessed.

Our study was a limited sample due to the COVID-19 pandemic which ended our data collection. As a result, we were not able to acquire complete datasets (through skilled reaching) for half of our infants who were already enrolled. Despite our limited sample size, our data are important as they reflect the variability that is commonly observed in outcomes for infants born preterm. A subset of the infants in our study showed changes in relative power and coherence similar to infants with TD while others in our study were not similar. This variability in the pattern of brain function development is representative in a group of infants born preterm, who may or may not go onto develop neuromotor impairments. We do not expect different findings even in a larger sample; a consistent group pattern is not expected to emerge from a group of infants born preterm who appear to have a multimodal distribution (some preterm infants look like infants with TD while others do not). However, by utilizing the complete dataset to be collected, a more objective profiling of diverse subtypes of neural function changing patterns can be achieved with data driven algorithms (such as latent class analysis, and cluster analysis), which will be one of our future efforts.

The higher variance in gradients, intercepts, and fitting errors indicate that the developmental heterogeneity is larger in the preterm group, compared to the TD group (see Table 2). The larger heterogeneity supports our findings that our preterm group shows mixed trajectories leading to no group differences when compared to the TD group. Our individual-level analyses further supported the increased heterogeneity in the preterm group compared to the TD group from our previous dataset. The higher variance in our current dataset demonstrates that the predictors (age, reaching skill level, and fine and gross raw motor scores) used in the previous study examining infants with TD do not play a clear, consistent role in the present study’s dataset. This suggests that there was no significant and alpha-band specific correlation with age in our sample. Furthermore, our sample of infants born preterm represent the variability typically seen in this population. As such, individual analyses compared against infants with TD provides meaningful insight into future analyses that would improve our understanding of changes in neural function as infants born preterm learn to motor skills.

EEG has known limitations such as sensitivity to non-neural factors like skull and tissue thickness between the cerebral cortex and electrodes. Moreover, noise introduced by factors such as hair and the fit of the cap also introduce artifacts into the data. We adopted a comprehensive data preprocessing pipeline and used relative power instead of absolute power to normalize the data within individual sessions, thereby mitigating these issues. Additionally, we employed likelihood ratio tests that consisted of two models, a null and alternative, to test the effect of subject variation (e.g. repeated measurements). In the null model, the random intercept was removed and compared to the alternative model, which consisted the random intercept. This test showed that there was no significant impact due to subject variation (*p* = 0.07).

EEG referencing is another point of debate. We used cortical reference points for EEG processing similar to other studies examining infant motor development. This allows us to make direct comparisons between our results with previous studies, which is an important element as our goal is to better understand the differences between infants born preterm and term. EEG amplifiers measure potential differences between activities recorded by two electrodes; thus, the reference electrode is ideally electrophysiologically silent^18,19^. Commonly used references are the ear, linked mastoids, vertex, and neck ring, but unfortunately no site is truly inactive^18,19^. Alternative approaches such as the reference electrode standardization technique (REST) recovers the temporal information in EEG by standardizing the reference point at infinity^20^. As studies continue to show the impact the choice of reference point has on the EEG analysis, future research will need to compare and contrast findings that utilize methods such as REST^20^ with our current selection.

In conclusion, our current study showed that some infants born preterm showed developmental patterns of brain function similar to infants with TD, while others did not. How these findings related to neurodevelopmental outcomes is unknown. Future research is warranted into examining different frequency bands in this population to explore the relationship with emerging arm reaching skill. This study was an important first step to characterize the neural function of infants born preterm as arm reaching emerges. We hope that future studies can build off of ours to provide further insight onto determining which infants born preterm go onto develop neuromotor impairments.

## Method

### Participants

This study was approved by the Institutional Review Board of the University of Southern California and all methods were carried out in accordance with relevant guidelines and regulations. Informed consent was obtained from a parent or legal guardian before participation. Infants were recruited from local hospitals in the Greater Los Angeles area of California or word of mouth and were eligible to enroll if their gestational age at birth was less than 32 weeks. Infants with unstable medical conditions were excluded.

Fourteen infants between the adjusted ages of 56 and 295 days participated in the study. Adjusted age was calculated by subtracting the number of days between due date and date of birth from the chronological age in days at the time of the visit. Our goal was to measure infants longitudinally across the time period when arm reaching was emerging. Monthly visits ranged from 1 to 5 and varied for each infant. Monthly visits started as early as possible and continued until the infant demonstrated skilled reaching, determined by a research team member. The total dataset had 7 participants with 3–5 sessions, 3 participants with 2 sessions and 4 participants with 1 session. Data collection was stopped in March of 2020 due to the COVID-19 pandemic. This resulted in 7 participants with complete datasets (through skilled reaching) and 7 participants with partial datasets. This resulted in a total of 37 EEG sessions for preprocessing and analysis.

### Session overview

At each visit, a wearable sensor (Opal sensor; APDM Inc, Portland, Oregon) was placed on each arm of the infant using a custom wrist band with an internal pocket. Infants were video recorded for five minutes of spontaneous movements in supine position. The infants wore these sensors for the remainder of that day as they went about their typical activities. Sensors were collected by a research team member on a later date. Next, infants were fitted with an EEG cap based on head measurements taken at the beginning of each session. After fitting the cap, grommets were filled with water-based gel (Signa Gel, Fairfield, New Jersey, USA) before electrodes were secured into place. Electrode offset-values were under 20 kΩ using the ActiView acquisition software (version 7.05, BioSemi, Netherlands). EEG data were recorded using a Biosemi system (BioSemi, Netherlands) with 32-electrodes at a sampling rate of 2048 Hz with a time-synchronized web camera integrated using the MotionMonitor software (version 9.18, Innovative Sports Training Inc., Chicago, IL, USA). Additionally, a video camera (Sony Handycam, New York, NY, USA) on a tripod recorded the session from the opposite side from the web camera. The first three trials were 20-s resting state baseline trials, followed by a minimum of nine 20-s reaching trials, and followed by three 20-s baseline trials. During baseline trials, a researcher presented a glowing globe toy beyond the infant’s reach to attract to attract their visual attention and minimize body movements. During reaching trials, the researcher presented a toy, within reach and at midline, encouraging the infant to reach for the toy. After a successful reach, the researcher allowed the infant to play with the toy briefly and then retrieved the toy to present it again. This was repeated throughout the duration of the reaching trials. EEG data during reaching trials are not presented here. The purpose of the present study was to investigate EEG data during resting state in infants born preterm. Following the EEG session, we administered the BSID-III^12^, and took anthropometric measurements (see Table 4). The BSID-III includes the assessment of cognitive, language, and motor development. This was administered by a research team member and took approximately 25–30 min to complete per visit.

**Table 4:** Participant characteristics grouped by month and shown as minimum and maximum values for each measurement.

Group (number of sessions)	Age (days)		Weight (kg)		Length (cm)		Head Circumference (cm)		FMS (raw score)		GMS (raw score)	
	Min	Max	Min	Max	Min	Max	Min	Max	Min	Max	Min	Max
Month 2 (4)	56	74	4.65	6.1	52	58	36.5	39.5	6	8	8	9
Month 3 (8)	84	111	5.15	6.4	55.5	60	38.5	40.5	5	12	11	16
Month 4 (7)	122	139	5.84	7.83	60	63	39.5	43.5	10	18	13	20
Month 5 (7)	142	167	5.84	8.52	59	65	40.5	44	7	17	14	27
Month 6 (5)	170	195	6.7	8.84	63	69	41	45.5	8	18	16	20
Month 7 (2)	196	211	6.32	8.56	64	70	41.5	46	17	20	22	26
Month 8 (2)	233	245	7.15	9.71	65	72	43	47.25	20	21	17	29
Month 9 (1)	260	260	7.6	7.6	66.5	66.5	44	44	20	20	16	16
Month 10 (1)	295	295	7.7	7.7	69	69	44	44	22	22	23	23
All (37)	56	295	4.65	9.71	52	72	36.5	47.25	5	22	8	29

Group months 9 and 10 contained 1 dataset, thus minimum and maximum are the same values. Age are in days adjusted for premature birth. Fine motor scores (FMS) and gross motor scores (GMS) are raw scores from the fine and gross motor section of the Bayley Scales of Infant Development— III.

Group months 9 and 10 contained 1 dataset, thus minimum and maximum are the same values. Age are in days adjusted for premature birth. Fine motor scores (FMS) and gross motor scores (GMS) are raw scores from the fine and gross motor section of the Bayley Scales of Infant Development—III.

### Behavior coding

We used the synchronized video to identify undesired behaviors during the baseline trials, which would allow us to remove the corresponding EEG segments. This allowed us to process EEG data that reflected a baseline (resting-state) condition. Undesired behaviors included movements that represented fussiness such as repeated arm, leg, and trunk movements, and movements of the head and body that caused artifacts in the EEG data. Additionally, research team member assessed the reaching skill level at each session to assign one of four reaching skill levels for each infant (1 = no reaching; 2 = low skill, moves arm towards toy with no grasp; 3 = medium skill, moves arm towards toy with indirect path, pre-shaped hand without grasp; 4 = high skill, moves arm directly towards toy and grasps with pre-shaped hand.

### EEG preprocessing

The Biosemi system automatically references each electrode to the common mode sense (CMS) active electrode, and common mode information exists in the recorded signals. EEG data were re-referenced to the average of T7 and T8 electrodes based on the international 10–20 system. This re-reference steps helps complete the systems’ full 80 dB common-mode rejection ratio (CMRR). Next, to remove direct current off-set and interference from high frequency components (e.g. powerline noise etc.), we employed a bandpass infinite impulse filter (0.3–30 Hz) to the re-referenced data. We selected this frequency range to include infant alpha activities within 6–9 Hz, while reducing effects from other sources. Next, segments with large fluctuations were visually identified and the corresponding times were noted for each session. We then merged these times with the time segments of the undesired behaviors from the aforementioned behavior coding to construct a table of start and end times for each unwanted segment identified. Using EEGLAB, we removed these segments from the EEG time series and concatenated the remaining segments for subsequent analyses. After bad segment rejection, the range of EEG data was between 12.4 and 94.8 s, with a mean duration of 47.6 s and a standard deviation of 22.3 s. Next, Kurtosis indices were calculated for all electrodes. Electrodes with Kurtosis indices beyond the five standard deviations of all electrodes were noted and rejected. The data from the rejected electrode(s) were interpolated by the average of surrounding electrodes. Next, we applied a common average reference by re-referencing each electrode to the average of all electrodes to spatially filter out common-mode artifacts. Finally, an independent component analysis, using EEGLAB’s *runica* was used to decompose EEG data into independent components (IC) from brain sources and unwanted artifacts. Components caused by eye movements, electrocardiography, and motion artifacts were visually identified and removed from further analyses. With a total of 32 possible ICA components, a minimum of 10 and a maximum of 29, with an average of 22.38 ± 5.37 components were rejected. Each IC was closely examined by inspecting the temporal, spectral, and spatial patterns. All preprocessing steps were performed using the MATLAB-based toolbox EEGLAB v13 software^21^.

### Spectral analysis (relative power in the alpha band)

To study changes across time in alpha activities from infant EEG, we calculated power spectral densities (PSD) using Welch’s method, or “pwelch” function in MATLAB (ver. 2019a, MathWorks Inc., Natick, MA., USA). For PSD estimation, we chose a 2-s Hann window with a 50% overlap between segments. This resulted in a frequency resolution of 0.5 Hz to capture the changes in spectral activities in our EEG data. We then transformed PSDs into relative powers to allow for comparisons across sessions. To calculate relative power, we divided PSD by the sum PSD from all bins for each frequency bin within 0 and 30 Hz and each electrode. Alpha band powers from motor cortices were calculated for each session by averaging activities from electrodes C3, C4, and Cz, and summing the relative powers of all bins within 6–9 Hz. To provide a qualitative view of changes along age, sessions with the same monthly age were grouped and their alpha activities from the motor cortices were averaged. Additionally, to examine developmental changes of alpha activities in the spatial domain, we constructed topography plots by registering the monthly-averaged alpha band powers from each electrode to their geodesic locations on the scalp. These maps were visually inspected to determine any developmental trends based on age.

### Connectivity analysis (coherence)

To study the changes in the alpha activities between brain regions, we evaluated connectivity which was based on the coherence values between electrode pairs. Coherence values were calculated using the *mscohere* function in EEGLAB/MATLAB. This function is based on Welch’s method to estimate PSD for each electrode of each electrode pair as well as cross-electrode PSD^22^.

1 $$Coh_{E1E2} \left( f \right) = \frac{{PSD_{E1E2} \left( f \right)^{2} }}{{PSD_{E1} \left( f \right) \times PSD_{E2} \left( f \right)}}$$

*Coh*_*E1E2*_*(f)* is the magnitude-squared coherence between electrode *E*_*1*_ and *E*_*2*_ at frequency *f*. *PSD*_*E1*_ and *PSD*_*E2*_ are the corresponding PSD estimations, and *PSD*_*E1E2*_ are the cross-electrode PSD estimation. Hann window with a length of 2 s and 50% overlap were chosen in the PSD estimation to maintain consistency with our spectral analysis. Mean alpha coherence was defined as the average of coherences from all bins within 6–9 Hz. A total of 18 pairs of electrodes were chosen to examine changes in connectivity across the functional brain regions, including frontal, motor, and parietal cortices (see Table 2 for list of pairs). These pairs were chosen based on our group’s previous study^11^ which investigated the same electrode pairs and brain regions.

### Comparison of variance in developmental heterogeneity

To demonstrate the difference in variability between infants with TD and born preterm, participants with more than two visits were identified and subject to the following analysis. For the TD group, we used the same dataset as our previous study investigating the neuromotor development of infants with typical development^11^. The dataset contains 71 EEG recording sessions from 21 participants between the age of 1 to 7 months. After removing single-session participants, there were 10 participants left in infants born preterm and 18 participants left in the TD group. Linear fitting of motor alpha band power against age was performed within each participant across their respective visits. To quantify the variation of developmental trajectories, we first extracted gradient, intercept, and fitting error for each fitted line. Then, the variances of these metrics were calculated to evaluate various aspects of the developmental heterogeneity in both groups. The gradient represents the rate of change in motor alpha band power as a function of age, so its variance captures the variation in alpha power progression among individuals. Similarly, variance of intercepts represents cross-individual variability at baseline time. Fitting error represents the vertical deviations from each datapoint to the fitted line. Thus, its variance accounts for within-individual developmental fluctuations along age. With these three metrics, we can describe developmental heterogeneity in our sample of infants born preterm and subsequently compare it with the TD group.

### Statistical analyses

#### Relative power

To test if relative power of the alpha band increased as reaching skill emerged in infants born preterm, we employed a Pearson correlation test between relative power and age (*p* < 0.05). Since there were multiple samples from the same participants, we employed a likelihood ratio test by constructing two linear mixed-effects model (LMM) to address the non-independency within the grouping factor (participants). One model with and another without subject variation as a random effect were compared. This comparison informed us regarding the impact of subject variation in our dataset. A significant difference in this comparison would indicate that this relationship is not as informative due to repeated measures from the same participants.

To further validate the relationship between alpha band power and age discovered under a limited sample size, we took a bootstrapping approach to establish a null distribution of correlations between randomly generated motor alpha band power and age. Specifically, frequency bins between 0 and 30 Hz were randomly shuffled 100 times. After each shuffle a pseudo-alpha power were calculated and correlated to age, thus producing 100 correlation coefficients. These correlations were then compared to our true correlation between motor alpha band power and age. This comparison tests the motor alpha-band specific correlation with age in our sample of infants born preterm against random.

#### Brain connectivity (coherence)

To examine if changes in coherence values had a significant relationship with age and motor development scores, we used a Pearson correlation test. We tested the correlation of mean alpha coherence from each of the 18 electrode pairs against age and motor development scores (reaching skill scores, BSID-III raw fine motor subscale scores, BSID-III raw gross motor subscale score). We included age and three measures of motor skill as we expected all of them would be related (they are not independent measures) and we wanted to examine which relationships were stronger. Infants develop at different rates; thus, infants of the same age are typically different points in terms of their reaching skill and/or overall motor development. If coherence values are related to neuromotor development, then we would expect a stronger relationship between coherence values and measures of motor skill than we would between coherence values and age. Finally, there were repeated measures from most of our participants, making simple linear regression not appropriate. Thus, we employed the LMM to model the relationship between coherence and predictors (age and motor development scores). Separate LMM models were created for each predictor as they possessed unique intrinsic characteristics (Eqs. 2–5). A significant difference in these two models would indicate that the predictor plays a role in its relationship with coherence, whereas a non-significant difference indicates that the predictor does not play a role in its relationship with coherence.

2 $$Coh{ }\sim { }Age + \left( {1{|}Participant} \right) + \varepsilon { }$$

3 $$Coh{ }\sim { }Reaching\,Skill\,Level + \left( {1 + Reaching\,Skill\,Level{|}Participant} \right) + \varepsilon { }$$

4 $$Coh{ }\sim { }FMS + \left( {1 + FMS{|}Participant} \right) + \varepsilon$$

5 $$Coh{ }\sim { }GMS + \left( {1 + GMS{|}Participant} \right) + \varepsilon { }$$

*Coh* represents the mean alpha coherence; *Age* represents adjusted age in days at the time of visit; Reaching Skill Level, FMS (fine motor scores), and GMS (gross motor scores) denote the motor developmental scores. The equations were expressed in Wilkinson notation. ε is the error term. *(1|Participant)* refers to the random intercepts within the grouping factor (participants) to account for repeated measures, which was added in all equations. For Reaching Skill Level, FMS, and GMS, the random slope terms were also added to account for individual progression variance of these metrics presented in the grouping factor (participants), as shown in Eqs. (3–5).

To test if predictors presented a significant effect on the changes of coherence, likelihood ratio tests were performed on two LMM models for each predictor. This was achieved by removing the targeted predictor from the original model as null models and comparing their goodness of fit to the original equation (Eqs. (2–5)). A significant difference in these two models would indicate that the predictor plays a role in its relationship with coherence, whereas a non-significant difference indicates that the predictor does not play a role in its relationship with coherence. For both Pearson correlation tests and likelihood ratio tests, significance level was set at 0.05 and was adjusted using the Bonferroni correction for multiple comparisons (i.e. 0.05/18). For predictors, age and fine and gross motor scores, scatter plots with least-squares fitted lines were created to show the changing trends and correlation coefficients were calculated to quantify the relationship with mean alpha coherence for each electrode pair. Due to sparse sample points, bar plots with standard deviation were chosen to present changes in mean alpha coherence across the different reaching skill levels.
